# Value of Veratrum as a Prophylactic against Peritonitis after Surgical Operation Involving the Opening of the Peritoneal Cavity

**Published:** 1884-12

**Authors:** James Roane

**Affiliations:** Yankton, Dak.


### VALUE OF VERATRUM VIRIDE AS A PROPHYLACTIC AGAINST PERITONITIS AFTER SURGICAL OPERATIONS INVOLVING THE OPENING OF THE PERITONEAL CAVITY.

BY JAMES ROANE, M.D., YANKTON, DAK.

On August 25th, assisted by Drs. D. F. Etter and W. H. Turkopp, I operated upon R. S———, a short and spare Bohemian, aged fifty-five, for the relief of strangulated hernia (right, complete oblique inguinal). A careful and thorough trial of the taxis, at first without the aid of anæsthetics, and subsequently when they were administered to the surgical extent, had failed to produce reduction. Owing to the urgency of the symptoms, the operation was performed at night, and was more protracted than it would otherwise have been on account of the very poor light of a single lamp. The intestine was found to be deeply congested, but showed no signs of disorganization. The constriction was at the internal ring, and its division was followed by the escape of about two ounces of sanguinous serum. The hernia having been reduced, the upper three-fourths of the incision were closed by sutures, and the lower fourth left open for drainage. Throughout its various steps the operation was conducted under strictly antiseptic precautions—a solution of corrosive sublimate, one to two thousand, being used for that purpose. A pledget of absorbent cotton, soaked in this solution, was placed over the wound; this was covered by a larger pad of cotton, and the whole was held securely *in situ* by a few turns of a roller bandage. On coming from under the influence of the anæsthetic the patient vomited stercoraceous matter profusely. He was put to bed, given one-fourth of a grain of morphia hypodermically, and placed upon one minim of Norwood's tincture of veratrum viride, and ten minims of deodorized tincture of opium, every two hours—day and night. This treatment was followed up to the morning of the 28th, when it was discontinued because of the fall of the temperature, the total absence of any of the signs of peritonitis, and the increasing prostration of the patient.

The physiological action of the veratrum viride at this time was very marked, manifesting itself in the sense of extreme muscular weakness, and in the slow, moderately full, but soft pulse, which became very rapid on the patient's making the least exertion. At no time after the operation were there any of the local signs of peritonitis, such as pain, tenderness, or tympanitis. The highest temperature (taken under the tongue) was 100 8° ; and this occurred on the evening of the 26th. On the 29th the temperature was normal, and subsequently it did not rise above 99°. In that portion of the incision which was closed by means of sutures, union took place by first intention. A slight serous discharge oozed from the lower fourth, left open for drainage for forty hours, but thereafter no stains could be detected on the cotton dressing, and the wound rapidly closed. On September 9th a well-fitting truss was applied, and the patient discharged.—*N. Y. Med. Record.*

